# A near real-time framework for monitoring very-long-period signals at volcanoes

**DOI:** 10.1038/s41598-025-25636-7

**Published:** 2025-11-24

**Authors:** Sergio Gammaldi, Dario Delle Donne, Pasquale Cantiello, Antonella Bobbio, Teresa Caputo, Walter De Cesare, Antonietta M. Esposito, Rosario Peluso, Massimo Orazi

**Affiliations:** Istituto Nazionale di Geofisica e Vulcanologia (INGV), Sezione Osservatorio Vesuviano, Napoli, Italy

**Keywords:** Natural hazards, Solid Earth sciences

## Abstract

Real-time seismological applications are essential for monitoring active volcanoes, offering valuable tools for the early detection of volcanic unrest and eruption. Very Long Period (VLP) seismicity, commonly observed at open-vent volcanoes with mild and persistent explosive activity, is a key indicator of volcanic activity intensity as changes in the rate of occurrence and VLP event magnitude can be a signal of impending unrest. In this study, we introduce a new method for the automatic and near real-time detection and characterization of VLP seismicity. Our approach was tested on Stromboli Volcano (Italy), where VLP seismic activity has been well-documented for over two decades. The detection algorithm is based on three-component amplitude analysis, derived from waveform polarization and spectral characteristics of continuous seismic records. It extracts key parameters such as detection time, event duration, azimuth, and incidence (polarization) angles. VLP events are distinguished from other signals through a single-station statistical analysis of polarization parameters, providing a reliable near–real-time catalog of VLP detections. Optimal detection thresholds for each station were determined using a machine-learning hyperparameter optimization approach. Here, we focus on the year 2007, which was characterized by highly variable VLP activity, including a major effusive eruption at Stromboli. The algorithm’s performance was validated using an independent, manually inspected dataset from 2007, yielding a false alert rate of 23% and a missed alert rate of 27% for the best-performing station. The results show that the method accurately reproduces the temporal evolution of the different activity phases throughout the year, with clear implications for enhancing and integrating VLP detection into existing volcano monitoring strategies. We applied the method to 16 years of seismic data (2009–2024), successfully reconstructing the temporal evolution of the VLP event rate in close agreement with manual inspections. The automatic detections show a strong correlation with manually derived daily rates, demonstrating that our automatic VLP detection time series reliably captures long-term fluctuations in volcanic activity over the entire period of investigation.

**Supplementary Information:**

The online version contains supplementary material available at 10.1038/s41598-025-25636-7.

## Introduction

The effectiveness of volcano monitoring is measured by how promptly it can provide authorities with indications of an impending hazardous phase. For this reason, volcanologists continuously monitor a range of parameters that, over time, have shown variations systematically associated with the onset of paroxysmal activity and other potentially dangerous conditions for the population. The occurrence of Very Long Period (VLP) events is a key seismic parameter at many open-vent volcanoes, which convey information about shallow conduit processes^1–4^. At open-vent volcanoes, VLPs typically originate within a few hundred meters of the crater and reflect the nucleation, ascent, and bursting of large gas slugs^5–8^, making them a valuable indicator of the intensity of magmatic degassing in the shallow portion of the feeding conduit. Despite their diagnostic value, monitoring VLP seismicity is not straightforward, as these low-frequency signals are often obscured by oceanic microseisms and other noise sources related to atmospheric phenomena. Multi-station approaches based on semblance analysis^9^, have been used for monitoring and characterizing VLP events^10^. However, these methods require multiple operational sensors to detect and localize events with sufficient reliability, an issue at active volcanoes, where maintenance is hindered by steep slopes and the explosive hazard faced by field technicians. As a result, monitoring networks often operate with only a limited number of functional stations, reducing the effectiveness of these techniques for monitoring purposes. This highlights the need for robust analysis techniques capable of extracting meaningful information even from single-station data, ensuring continuity in surveillance even under degraded operating conditions. Recent single-station machine-learning based methods have been developed to pick seismic P- and S-wave onsets continuously e.g^11–13^. These algorithms train on large global waveform datasets^14–17^, offering more automated and robust phase detection across diverse networks. However, these methods are primarily designed to detect higher-frequency signals, such as earthquake waveforms, and are not optimized to handle the low-frequency noise conditions that dominate the VLP band 0.01–0.2 Hz^18^. To address this technological gap, we present a single-station method for the near-real-time detection and characterization of VLP events, leveraging seismic attribute imaging techniques. Our approach integrates spectral and polarization analyses with the Three-Component Amplitude (TCA) metric^19,20^, incorporating the Polarization Angle Stability (PAS) metric^21^ to effectively distinguish genuine VLP signals from background noise and transient artifacts. Thresholds at the different stations were calibrated through a Supervised Data-Driven (SDD) optimization strategy implemented with Optuna^22^, using manual picks as the reference dataset.

We applied our method to the VLP seismicity of Stromboli, a persistently active volcano in the Aeolian Islands (Sicily) and one of the most intensively monitored open-vent systems worldwide. At Stromboli, VLP seismic activity serves as a key monitoring parameter, as both the rate and amplitude of VLP events have been observed to increase prior to more vigorous eruptive phases^23,24^. Conversely, a deepening of the VLP source during lateral effusive events has been interpreted as evidence of magma drainage following lateral eruptions^25,26^. The most common interpretation of the VLP source process at Stromboli involves an ‘elbow-shaped’ conduit segment, where large gas slugs ascend and impact the upper dike walls, generating VLP seismic pulses^2–4^. However, more recent studies propose a different mechanism, suggesting that VLP signals originate near the top of the magma column and are associated with conduit expansion/contraction cycles driven by the persistent mild Strombolian explosive activity^27–30^.

Since 2003, the dense seismic network operated by the Istituto Nazionale di Geofisica e Vulcanologia (INGV) has produced an extensive catalog of VLP events^10,30^. This unique long-term dataset provided a valuable opportunity to test our algorithm on a 16-year time series spanning from 2009 to 2024. The algorithm successfully reproduced the variations in VLP activity previously identified through manual inspection by seismic analysts, demonstrating its ability to capture even subtle fluctuations during ordinary explosive phases. Our algorithm represents a scalable and reliable tool for real-time VLP monitoring, enhancing the surveillance of Stromboli’s persistent mild, and occasionally paroxysmal, explosive activity. Thanks to its simplicity and effectiveness, we believe this method can be readily adapted to other open-vent volcanic systems exhibiting VLP seismicity, making it applicable to open-conduit volcanoes worldwide.

### The seismic network at stromboli of INGV-osservatorio Vesuviano

**Fig. 1:**
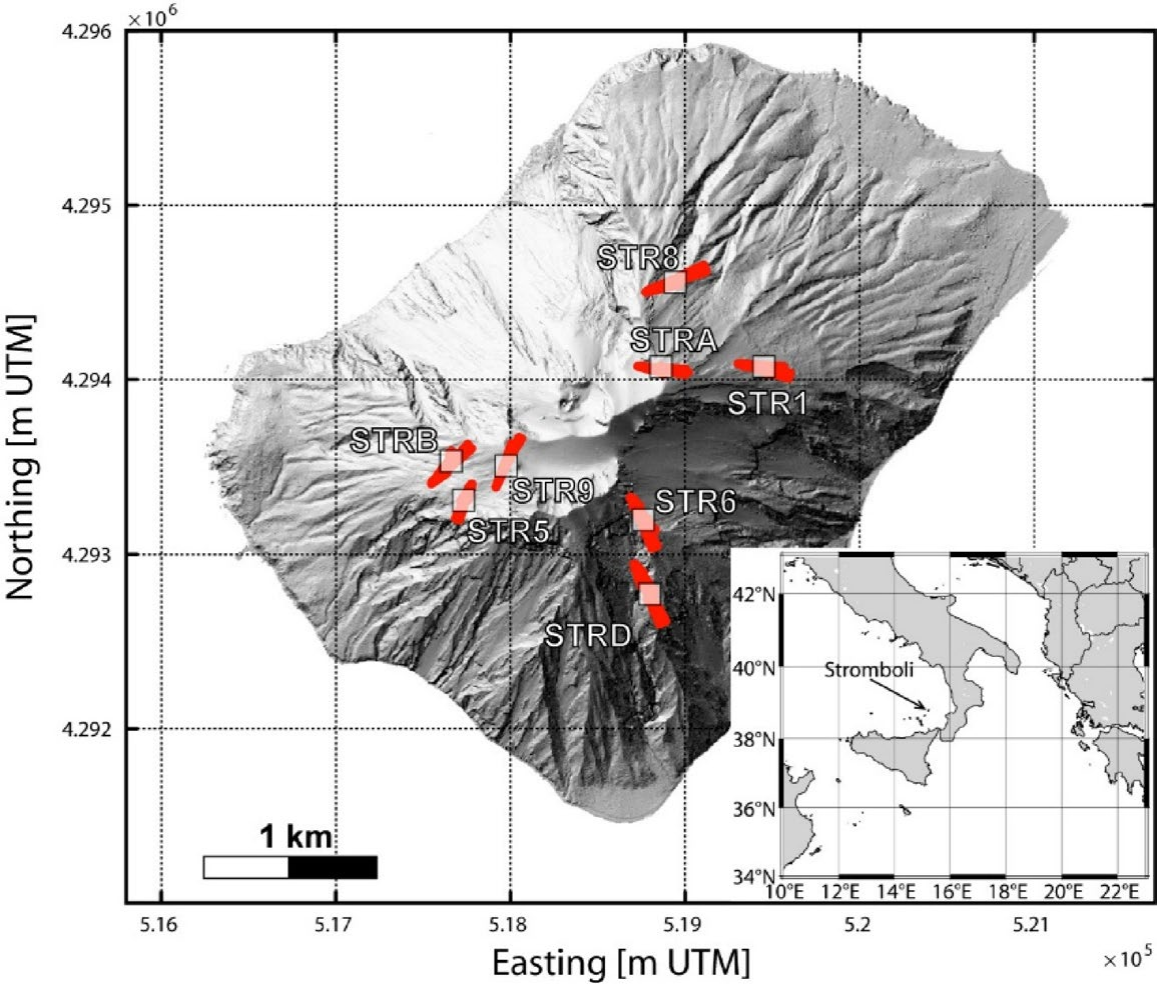
Shaded-relief map of Stromboli Volcano (Italy) showing the positions of seismic stations (white squares). Superimposed red bars indicate the average azimuths of VLP events automatically detected for the 2007. Coordinates are in UTM Zone 33 N; scale bar = 1 km. An inset (lower right) locates Stromboli in the southern Tyrrhenian Sea. The map was created using MATLAB Version: 9.9.0.1857802 (R2020b) Update 7 and Adobe Illustrator 29.6.1 1987–2025 Adobe.

The seismic network at Stromboli used in this study consists in 8 digital stations (Fig. 1) each equipped by a Guralp CMG 40 T broadband sensor recording at 50 Hz on 24 bit GILDA type digitizers^30,31^. Stations relay data via line of sight radio links to acquisition hubs on Stromboli and nearby Lipari, ensuring minimal latency. To boost sensitivity to the very long period band, during the recent years we have begun replacing the legacy CMG 40 T (60 s eigen period) instruments with Guralp 3ESPC seismometers, extending the usable band from 120 s up to 100 Hz. This upgrade not only sharpens our ability to detect and characterize VLP signals, but also supports remote sensor diagnostics and control, reducing the frequency of hazardous, high altitude maintenance trips (Fig. 1). As VLP signals originate at relatively shallow depths near or beneath the summit crater area^5^, we selected only the highest-elevation stations of the network, since the lower ones exhibit a signal-to-noise ratio too low for our purposes. The resulting sub-network includes eight digital stations, located at a minimum elevation of 500 m, covering approximately 180° of azimuth around the inferred VLP source area, with epicentral distances ranging up to 1500 m.

## Methods

Here, we present the confusion matrix (CM) test used as a tool to calibrate the optimal detection thresholds by comparing the algorithm’s results with manually picked events. Successively, we also outline the main procedural steps of our method which describe the algorithm and the data processing workflow used to extract key parameters associated with VLP waveforms, as well as details of the optimization strategy adopted to determine the most effective station-specific thresholds for VLP detection. A concise overview of the adopted workflow is provided in Table 1.

**Table 1:**
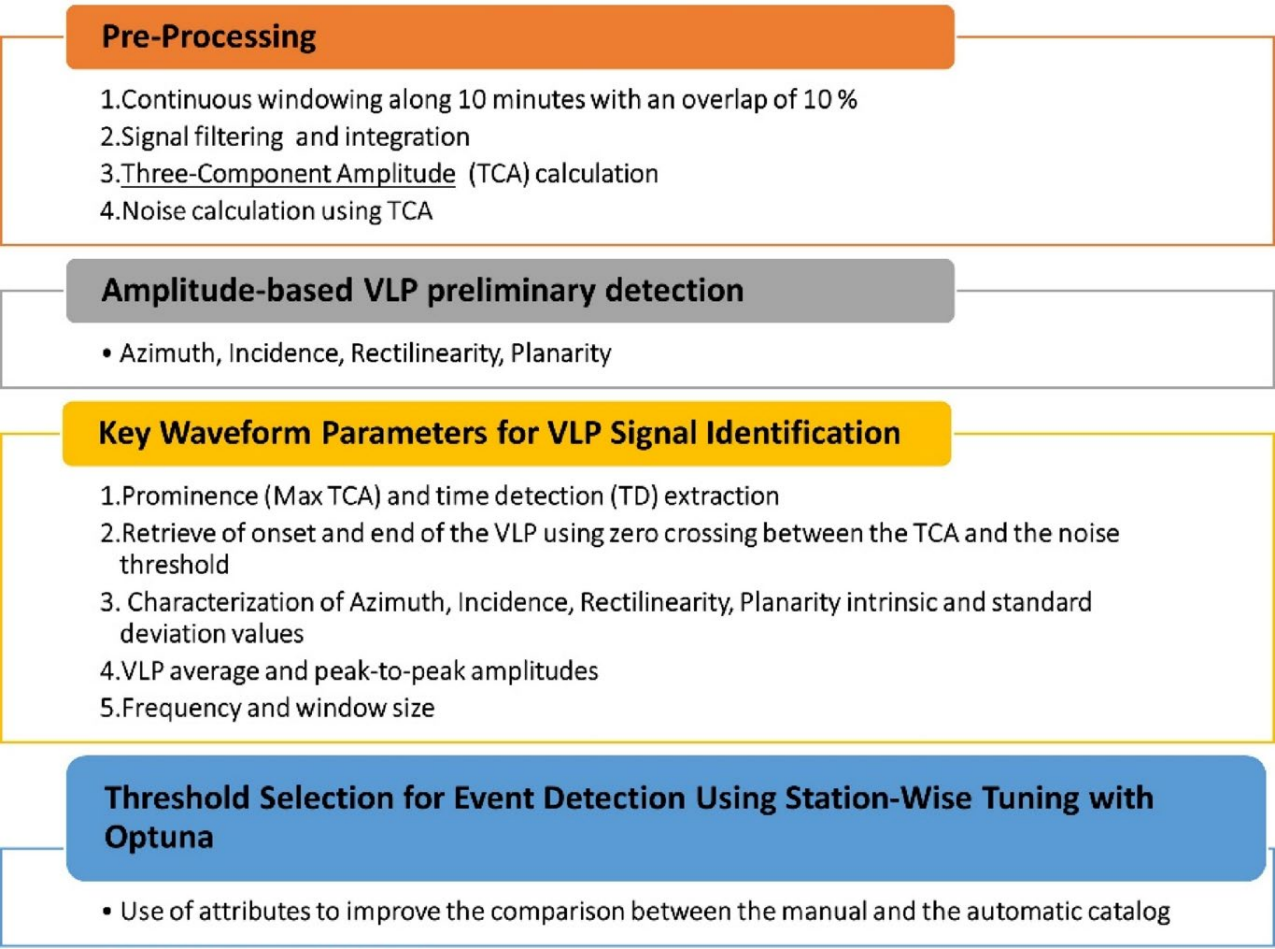
Methodology procedure steps: from the top to the bottom, the main step of the procedure deeply explained in Methods.

### Confusion matrix test

We selected the activity period encompassing the entire year 2007 as a test case for calibrating and validating our algorithm, as this interval was marked by significant changes in volcanic activity. We compared the VLP detections produced by our automatic algorithm with manually identified VLP onset times, as reported by seismic analysts, which served as the reference dataset. The aim was to assess the precision and reliability of the automated detection method, as well as its ability to capture key variations in volcanic activity. As part of the volcano surveillance activities coordinated by the Italian Civil Protection Agency, INGV – Osservatorio Vesuviano is responsible for monitoring VLP seismic activity at Stromboli volcano^25^. This monitoring is carried out daily by seismic analysts, who visually inspect bandpass-filtered helicorder recordings from all available stations. Based on this analysis, a daily VLP occurrence rate is computed to update the long-term VLP activity database and to support the comprehensive assessment of Stromboli’s activity state. This manually curated dataset served as our ground truth for evaluating the reliability of the proposed automatic detection method. The manual VLP database is typically based on the data from STRA station (Fig. 1), the station with the best signal-to-noise ratio for VLP signals. When STRA was unavailable, other stations in the network with the best signal-to-noise ratios were used to fill data gaps, ensuring continuity in the dataset. The method we used to compare the two datasets is the CM approach^32,33^. This approach relies on the parameters of true positives (TP), false negatives (FN), and false positives (FP), defined as follows: TP are VLPs that match the manual database; FN are VLPs missed by the automatic method; and FP are VLPs detected automatically but not present in the manual database.

For the matching criteria, a tolerance time window of ± 30 s was used to distinguish between TP, FN, and FP, accounting for variations in picking times across different stations, which may apply different filters and introduce variable delays. Consequently, VLP events occurring less than 60 s apart are considered as single events in this study. Next, the confusion matrix was constructed by calculating the precision and recall, two key metrics used to evaluate the performance of a classification model. Precision measures the accuracy of positive predictions. It indicates how many of the instances predicted as positive are actually positive. It is calculated as: PR=$\:\frac{TP}{TP+FP}$​A high precision means fewer false positives, ensuring that when the model predicts a positive class, it is likely correct. Recall (RC, also known as sensitivity) measures the model’s ability to correctly identify all actual positive instances. It is calculated as: RC=$\:\frac{TP}{TP+FN}$.

A high recall indicates fewer false negatives, ensuring that most true positive cases are detected by the model. A balance between precision and recall is often achieved using the F1-score, particularly when both metrics are equally important. This latter is calculated as $\:{F}_{1}score=2\frac{precision\times\:recall}{precision+recall}$​.

### Description of the algorithm

#### Pre-processing of data

**Fig. 2:**
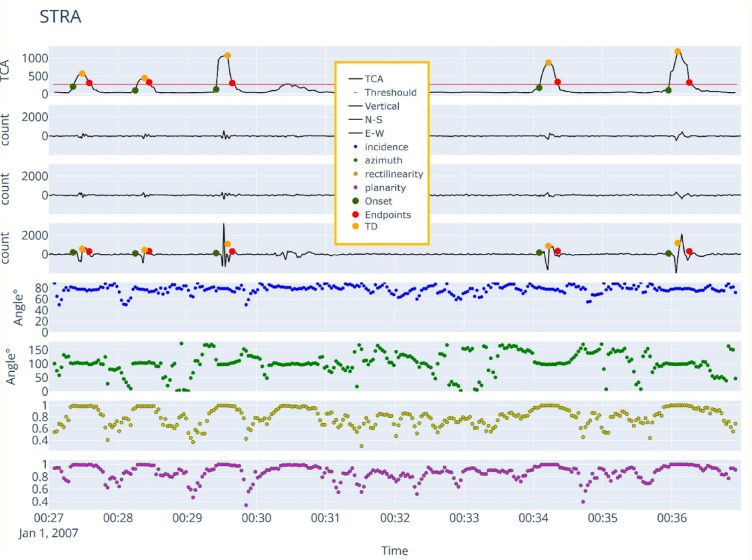
Example of a 10-minute-long, three-component seismic record from station STRA and the associated seismic attributes used in the detection algorithm. (**a**) TCA time history (in counts) with the noise threshold overlaid, which is used to define the onset, end, and peak amplitude of the event; (**b**), (**c**), and (**d**) show the three-component seismic record filtered in the VLP band (0.01–0.5 Hz); (**e**), (**f**), (**g**), and (**h**) display respectively the incidence angle, azimuth, rectilinearity, and planarity associated with the seismic record, highlighting the stability of polarization parameters during the VLP event compared to the surrounding ambient noise.

Prior to detection, continuous three component seismograms are subdivided into overlapping time windows to balance temporal resolution against computational load. Drawing on prior VLP analyses^25,26^, we selected a 10 min window length with 10% overlap (i.e., 1 min of overlap) to ensure that events straddling window boundaries are not missed (Table 1; Fig. 2b–d). Each window is band-pass filtered using a fourth-order Butterworth filter between 0.01 and 0.5 Hz, effectively suppressing higher-frequency noise while preserving the long-period content characteristic of VLP signals. The filtered velocity records are then numerically integrated to obtain ground displacement, which improves the signal-to-noise ratio and serves as the input to our automated detection algorithm. Finally, the Three-Component Amplitude (TCA) attribute is computed on each window to quantify the combined energy across the three sensor components, forming the basis for subsequent polarization and event-rate analyses. The Three-Component Amplitude (TCA) is defined as:

1 $$\:TCA=\sqrt{{\lambda\:}_{low}+{\lambda\:}_{med}+{\lambda\:}_{high}}$$

Where $\:{{\lambda\:}_{low},{\lambda\:}_{med},\lambda\:}_{high}$are respectively the smallest, intermediate, and largest eigenvalues of the three-component covariance matrix^19^. Specifically, for every 10 min data record, we compute TCA using a 10 s moving window with 20% overlap, leveraging ObsPy’s built-in *eigval* function^34^. To establish the dynamic noise threshold needed to extract the VLP signals, we calculate the root mean squared amplitude (RMS) of TCA over a 10 min sliding window (Fig. 2a). At the same time, we estimate the back-azimuth and incidence angles via eigenstructure decomposition^35^, following Vidale’s^36^ formulations (Table S1). These directional parameters help constrain source location and wave‐path geometry, aiding in the separation of VLP events from other seismic phases. We also computed other two polarization attributes, the rectilinearity and the planarity, using Jurkevics’s^19^ polarization analysis via ObsPy’s *flinn* routine. Rectilinearity quantifies the linearity of particle motion, with values close to 1 typically indicating body-wave arrivals, while planarity measures the degree to which motion lies within a plane, which is useful for identifying surface‐wave or anyway transversal polarized signals. Together with azimuth and incidence angles, rectilinearity and planarity form a robust feature set that significantly improves the fidelity of our automated VLP detection.

#### Amplitude-based VLP preliminary detection

To detect potential VLP events, we used the prominence of each local maximum in the TCA time history. Prominence, defined as the extent to which a given peak rises above the dynamically established noise threshold, is calculated as the difference between the noise level and the local maxima within a 10-minute window in the TCA time history (Fig. 2a). A potential VLP event is identified when the prominence exceeds a defined threshold, which has been carefully calibrated according to the specific characteristics of each station. Once a candidate VLP is detected, it is assigned a detection time (DT) and an amplitude value (Fig. 2). For each potential VLP event, the onset time and signal duration are determined by analyzing the zero-crossings between the TCA curve and the noise threshold (Fig. 2a). Within the event duration, we also extract the maximum TCA amplitude, the average squared amplitude, as well as the RMS amplitude for each component.

#### Key waveform parameters for VLP signal identification

**Fig. 3:**
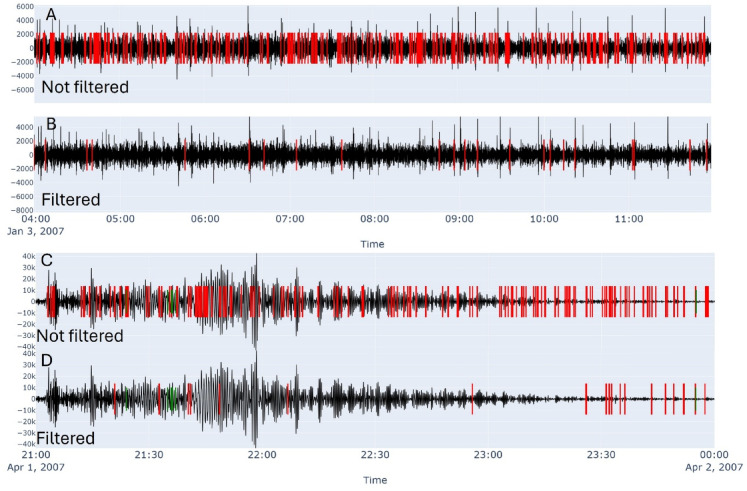
Two examples of PAS filtering using optimal thresholds identified with Optuna, applied during periods of elevated noise caused by a storm (**A**,** B**) and a teleseism (**C**,** D**). The results highlight the effectiveness of the filter in removing many preliminary VLP detections based solely on amplitude (a, c). In the supplementary material, all three components are shown in Fig. S1, S2, S5, and S6.

The VLP seismic band is contaminated by other natural sources, such as ocean microseism (Fig. 3a, Fig. S1) and teleseismic waves (Fig. 3c, Fig. S2), which can introduce false alerts into our database. Additionally, spikes, glitches, and gaps in the data stream may produce low-frequency artifacts, which could be mistakenly included in the database. However, manual inspection of the seismic traces allowed us to observe that, despite the presence of other low-frequency signals, VLP events can be distinguished by a relatively stable frequency and polarization angles throughout the entire duration (Fig. 2). By exploiting these distinctive features, our algorithm enables reliable discrimination of VLP events from other non-volcanic seismic signals. Therefore, for each preliminary VLP detection, we determine the frequency content using the short-time Fourier transform (STFT). Specifically, we compute a normalized spectrogram (see Fig. S3) using a Hanning window whose length is adapted to the duration of the selected VLP event, in order to evaluate frequency stability across the entire waveform. The dominant frequency of the potential VLP event is defined as the frequency bin with the maximum energy exceeding a normalized threshold of 0.9. Events that do not fall within the expected VLP frequency range or do not surpass this amplitude threshold are discarded, as they are considered unlikely to represent genuine VLP signals. Preliminary VLP detections were also associated with polarization parameters such as azimuth and incidence angles, rectilinearity, and planarity. These parameters were computed at 2-second intervals throughout the duration of each event, from which both the mean and standard deviation were derived. The standard deviation was then used to assess the stability of the polarization parameters (PAS)^21^, where low values indicate high stability and coherence, typical features of genuine VLP signals.

#### Threshold selection for event detection using station-wise tuning with optuna

To optimize VLP event detection, we implemented a station-specific tuning approach using the Optuna framework^22^. This optimization aimed to identify the optimal thresholds for signal amplitude and polarization parameters, including the standard deviation of azimuth and incidence angles, rectilinearity, and planarity, on a per-station basis. The objective was to minimize both false positives and false negatives, thereby enhancing the reliability of event classification across different stations. The heart of this optimization lies in the application of the Optuna hyperparameter optimization framework^22^. At its core, it employs Optuna’s Tree-structured Parzen Estimator (TPE) sampler, a sophisticated Bayesian optimization algorithm. This algorithm intelligently builds probabilistic models to distinguish between good and bad parameter regions, continuously refining its sampling approach. By maximizing the supervised F1-score, our system effectively “learns” the best filtering criteria for each station. This means it automatically adapts to the unique signal characteristics of each location, completely bypassing the time-consuming process of manual trial-and-error. To drive the Optuna pipeline, we engineered an objective function to generate trials, structured in the following manner:

1. Samples a candidate parameter set.
2. Application of PAS criteria selected by samples on each station’s VLP detections by these thresholds.
3. Retrieval of CM against the manual catalog, deriving precision, recall, and F1-score.

The optimization for each station was conducted independently by performing 150 trials per station (Fig. S4), meticulously exploring the 8-dimensional parameter space (Table 2). This process allowed us to define the optimal thresholds, the results of which are presented in this work. Overall, our PAS-based detection procedure, which incorporates such station-specific thresholds, allows us to filter out spurious events caused by adverse weather conditions characterized by low-frequency tremor in the VLP band, as well as low-frequency signals related to teleseismic arrivals from distant large earthquakes. This approach reduces false positives by approximately of the 15% for the STRA and up to 38% for STR5 if compared to the amplitude based preliminary detection (par. I), while retaining the majority of high-quality VLP detections (Fig. 3b and d, Fig. S5 and S6). This improvement underscores the inadequacy of amplitude-based methods for VLP detection in noisy volcanic environments and highlights the utility of polarization analysis for robust single-station monitoring.

## Results

### Quantitative assessment of the method on a year-long volcanic activity dataset

**Fig. 4:**
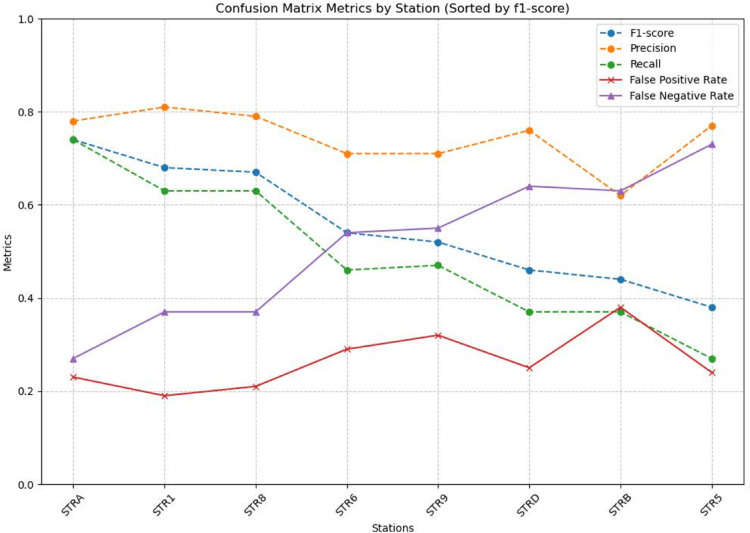
Confusion matrices for the test year 2007, ordered from left to right in decreasing F1-score. This order reflects the increasing distance of the stations from the summit craters (see Fig. 1). Although the F1-score decreases from 0.7 to 0.4 with increasing distance, consistent with the trend in recall, the precision remains roughly constant. This suggests that while a lower signal-to-noise ratio significantly affects the detection capability, it does not lead to an increase in false positives within the dataset.

The application of the CM to our dataset enables a quantitative evaluation of the method’s performance, based on the selection of optimal station-specific PAS threshold parameters using the SDD approach (see Methods, Fig. 4; Table 2). This also allows us to assess the detection reliability of every seismic station of the INGV network. We focused this evaluation on the 2007 dataset, which consists of 149,522 manually picked VLP waveforms serving as the ground truth for validation. The optimized detection thresholds indicate that most stations achieve a precision higher than 0.7 (except for STRB), reducing false alerts to 23% and missed alerts to 27% (Table 3). A precision value of 0.7 implies that approximately three out of ten detections are false, suggesting that, despite accounting for a wide range of parameters, the method still fails to completely eliminate spurious events. Among the network, station STRA (Fig. 1) shows the best performance, consistently achieving the highest F1-score and precision–recall metrics of 0.74, 0.78, and 0.74, respectively (Table 3).

We focused this evaluation on the 2007 dataset, which consists of 149,522 manually picked VLP waveforms serving as the ground truth for validation. The optimized detection thresholds indicate that most stations achieve a precision higher than 0.7 (except for STRB), reducing false alerts to 23% and missed alerts to 27% (Table 3). A precision value of 0.7 implies that approximately three out of ten detections are false, suggesting that, despite accounting for a wide range of parameters, the method still fails to completely eliminate spurious events. Among the network, station STRA (Fig. 1) shows the best performance, consistently achieving the highest F1-score and precision–recall metrics of 0.74, 0.78, and 0.74, respectively (Table 3).

As expected, the F1-score tends to increase with the station’s proximity to the crater area (Fig. 4), primarily driven by a reduction in false negatives (see the purple curve in Fig. 4). Precision remains consistently high across all stations, indicating that the algorithm reliably detects most real events identified manually, with few false positives. However, low-amplitude signals at more distant stations may be missed as the signal-to-noise ratio decreases with distance from the crater. Overall, Fig. 4 demonstrates that despite some variability in recall at remote stations, our method produces a robust VLP catalog with consistently low false alarm rates, supporting its suitability for long-term monitoring across the network.

**Table 2:**
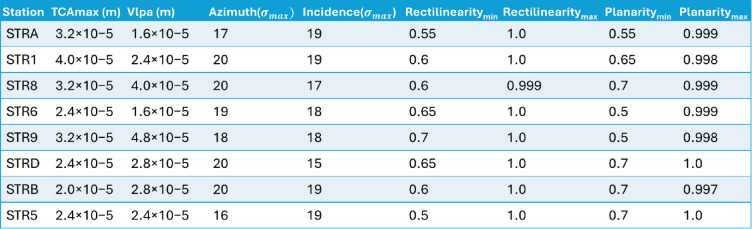
Best criteria threshold defined for each station according to the SDD method.

**Table 3:**
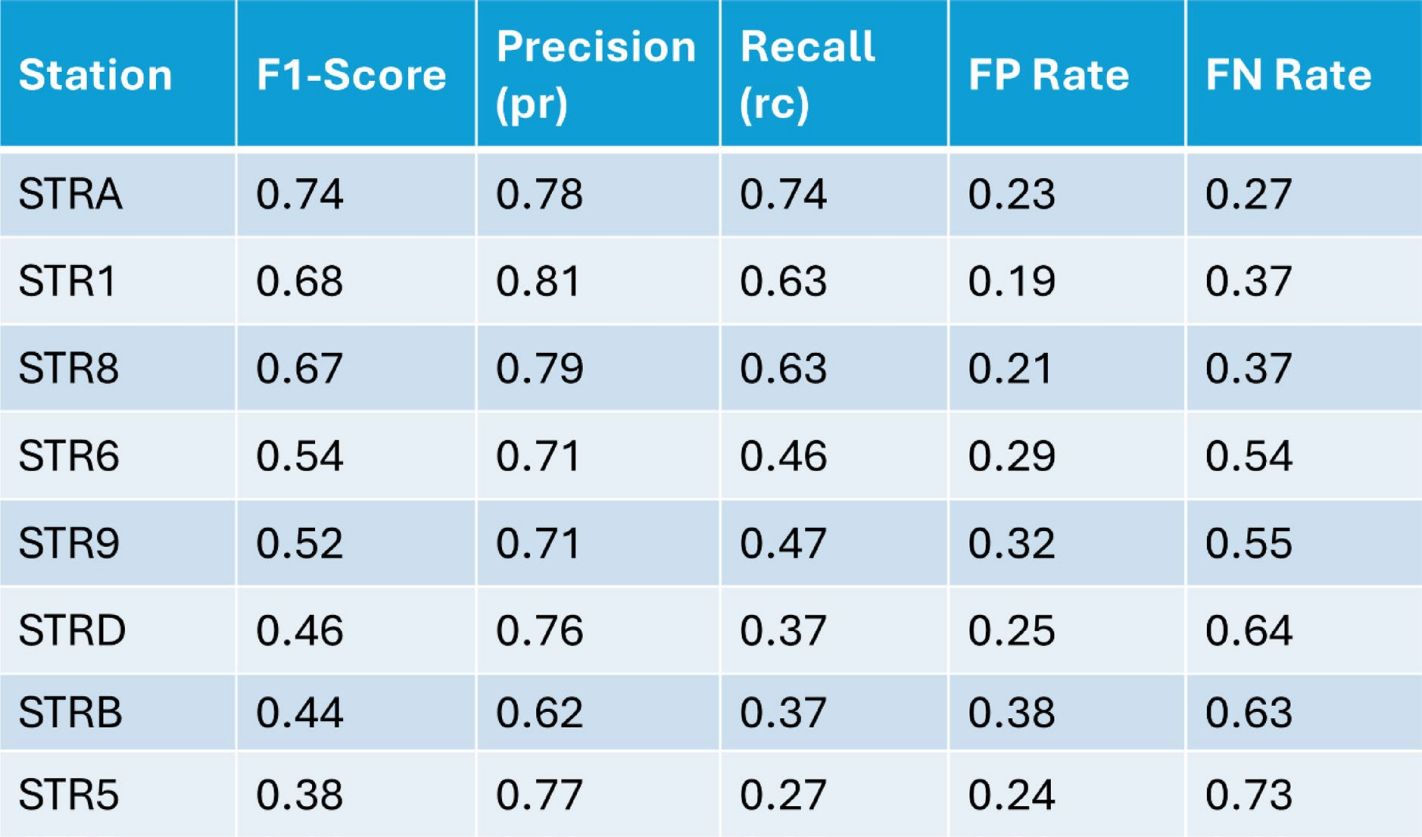
Confusion matrix values for the stations of the seismic network at Stromboli.

### Long-term fluctuations of VLP source position and event rate in the year 2007

In addition to providing the ground truth data used to validate the algorithm and to fine-tune the optimal station-specific thresholds, the year 2007 was marked by intense and variable volcanic activity^25^. This variability was also accompanied by significant changes in VLP seismicity, providing an ideal framework to experimentally test the sensitivity of our method to variations in eruptive behavior. In early 2007, Stromboli’s volcanic activity departed significantly from its typical Strombolian behavior, progressively intensified, culminating on 27 February with the opening of a new effusive lateral vent at approximately 400 m elevation on the Sciara del Fuoco^37^. Through this vent, shallow magma from the upper conduit drained over several days, resulting in a partial collapse of the crater terrace^23,38^. The lateral vent remained active through March and into early April 2007, emitting approximately 1–5 10^6^ m³ of lava^39^. This effusive phase was accompanied by a complete cessation of summit Strombolian explosions^40^, yet it was characterized by an exceptionally high rate of VLP seismicity, which peaked in April–May at 60–100 events per hour, unprecedented in prior records^25^. Notably, during this effusive activity, a paroxysmal explosive event occurred on 15 March 2007 at 20:38 UTC, producing a kilometer-high tephra column and ejecting meter-sized ballistic blocks down to 300 m a.s.l^39^. The effusive eruption waned over the course of April and ceased by the end of the month, while regular summit Strombolian activity gradually resumed thereafter. These dynamic eruptive changes were accompanied by significant variations in both VLP seismicity and polarization characteristics. Under normal Strombolian activity, VLP signals exhibit highly stable polarization angles, both azimuth and incidence, reflecting a steady source geometry^7^. However, during large effusive phases that involve substantial lava outpouring, these polarization angles can be significantly perturbed^41^. Giudicepietro et al.^25^ reported pronounced shifts in the azimuth and incidence angles of VLP events recorded at Stromboli, using the same stations employed in this study. They interpreted these variations as the result of rapid conduit drainage. Similarly, Ripepe et al.^18^ demonstrated that a progressive decrease in polarization angles corresponded to gravitationally driven magma-column withdrawal. These findings underscore the sensitivity of VLP polarization, particularly the incidence angle, as a reliable proxy for tracking magma-column depth and variations in the volcano’s activity state.

**Fig. 5:**
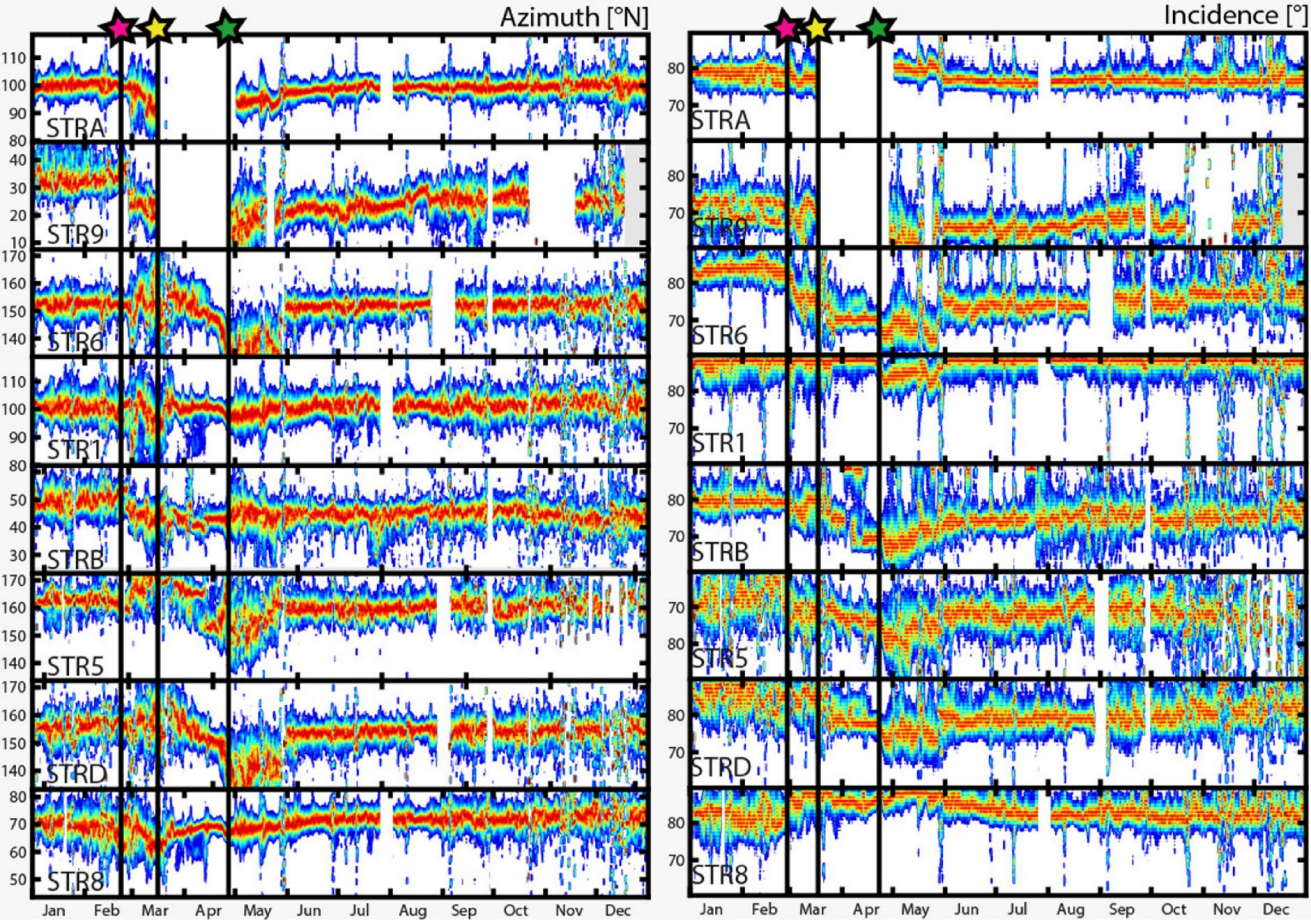
Temporal distribution of azimuth and incidence angles during the 2007 test year for each analyzed station. The figure provides insight into both^1^: the dominant azimuth and incidence values, represented by red tones that highlight the median of the respective distributions; and^2^ the variability in the estimates, where dark blue tones mark frequency bins with occurrence rates 30–60% lower than the most frequent bin (in red). Both panels indicate key eruptive phases: the onset of lava effusion on February 27, 2007 (red star), the timing of the paroxysmal explosion on March 15 (yellow star), and the end of the effusive activity (green star). Temporal trends in these parameters show significant changes following the onset of effusion, including a general decrease in incidence angle, suggesting a deepening of the VLP source and notable variations in azimuth, consistent with lateral migration of the source. After the eruption ends, the parameters return to stable, pre-eruptive values.

**Fig. 6:**
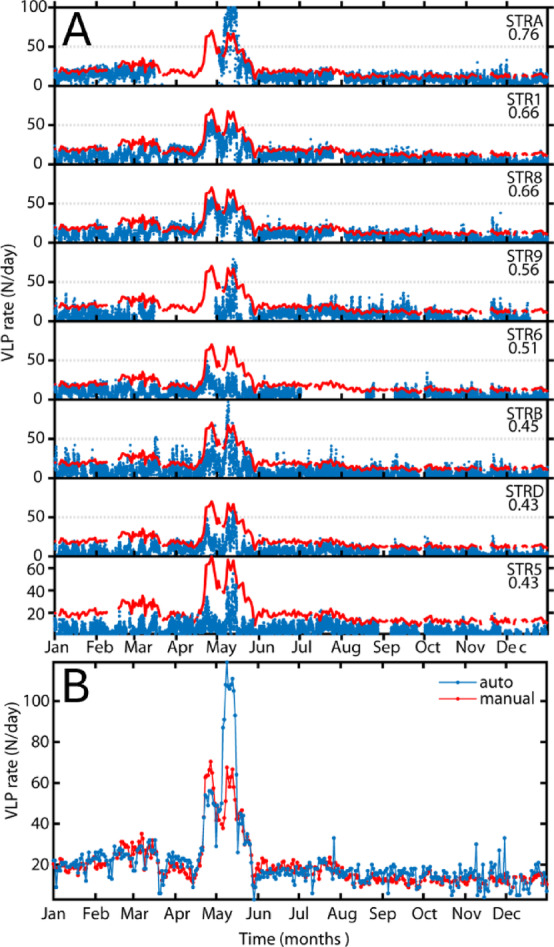
(**A**) VLP event rate estimated per station during the 2007 test year, evaluated on an hourly basis (blue dots), and compared with the manually determined daily VLP rate (red). Time series are ordered from top to bottom by decreasing F1-score. (**B**) Daily VLP event rate estimated from automatic detections using the station with the highest F1-score (blue), compared to the manual event rate (red). The temporal trends from the two datasets are highly consistent, although some discrepancies in absolute values appear during April–May, a period marked by a very high VLP rate but extremely low associated amplitudes.

Figure 5 presents the long-term trends of VLP azimuth and incidence angles derived from our algorithm for the entire year of 2007, which align well with the volcanic activity patterns previously described by Giudicepietro et al.^25^ and Ripepe et al.^26^. The parameters are shown station by station as 7-day averaged frequency distributions of the daily mean azimuth and incidence. This temporal averaging helps smooth out short-term fluctuations in volcanic activity and mitigates the influence of potential spurious measurements caused by non-volcanic phenomena or transient noise that may persist even after event filtering. The time evolution of azimuth and incidence angles effectively captures the same patterns described by Giudicepietro et al.^25^, while offering a clearer, more continuous perspective on their trends and correlations with volcanic processes. Specifically, azimuth and incidence show consistent behaviors across stations, remaining relatively stable on a monthly scale but exhibiting abrupt changes in response to the eruption onset (indicated by the red star). This disruption aligns with the drainage of shallow magma to approximately 400 m above sea level, the elevation at which the effusive vent opened^25,26,37^. Importantly, the pronounced variations in azimuth suggest that the VLP source migrated not only vertically but also laterally. This trend in source migration ceased abruptly following the paroxysmal explosion of 15 March 2007 (yellow star), during which ballistic ejecta destroyed the summit stations STRA and STR9. Despite this loss, the remaining stations continued to record the volcanic activity, highlighting the vulnerability of seismic instruments deployed in summit areas. Nonetheless, thanks to our single-station approach employed in this study, we were still able to track the ongoing activity using more distant sensors. After the paroxysmal phase, the VLP source appears to stabilize, gradually returning to pre-eruptive azimuthal values, while incidence remains either stable or continues its downward trend. The end of the eruptive phase (green star in Fig. 5), dated April 18, 2007^37^, is marked by an increase in incidence values, indicating a gradual rise of magma levels within the conduits, ultimately restoring persistent Strombolian activity at the summit craters (green star) (see Fig. 5). By the second half of the year, all parameters regain stability, reflecting a return to equilibrium in the volcanic system. We also tested the algorithm for calculating the VLP occurrence rate, a robust and rapid parameter commonly used to quantify the intensity of explosive volcanic activity, as they are related to the rate of magma degassing (Ripepe et al. 5 among others). As shown in Fig. 6, the VLP rates derived automatically closely match the manually determined rates in their long-term trends across all stations. At most sites, VLP activity peaks at 60–100 events per hour, consistent with the maxima identified through manual analysis. It is worth emphasizing that the automatic method operates on a single-station basis, and any data gaps caused by instrument failure are effectively mitigated by relying on the best-performing available stations, as determined by their F1-scores. In Fig. 6b, we present the VLP rates computed using the most reliable stations available at each time. These results show a strong correlation with the manually derived rates (Fig. S7), with an R² value of 0.76. Some discrepancies emerged during the peak of the volcanic crisis in May 2007, when exceptionally high VLP rates were recorded.

### Sixteen-year (2009–2024) long VLP event rate

**Fig. 7:**
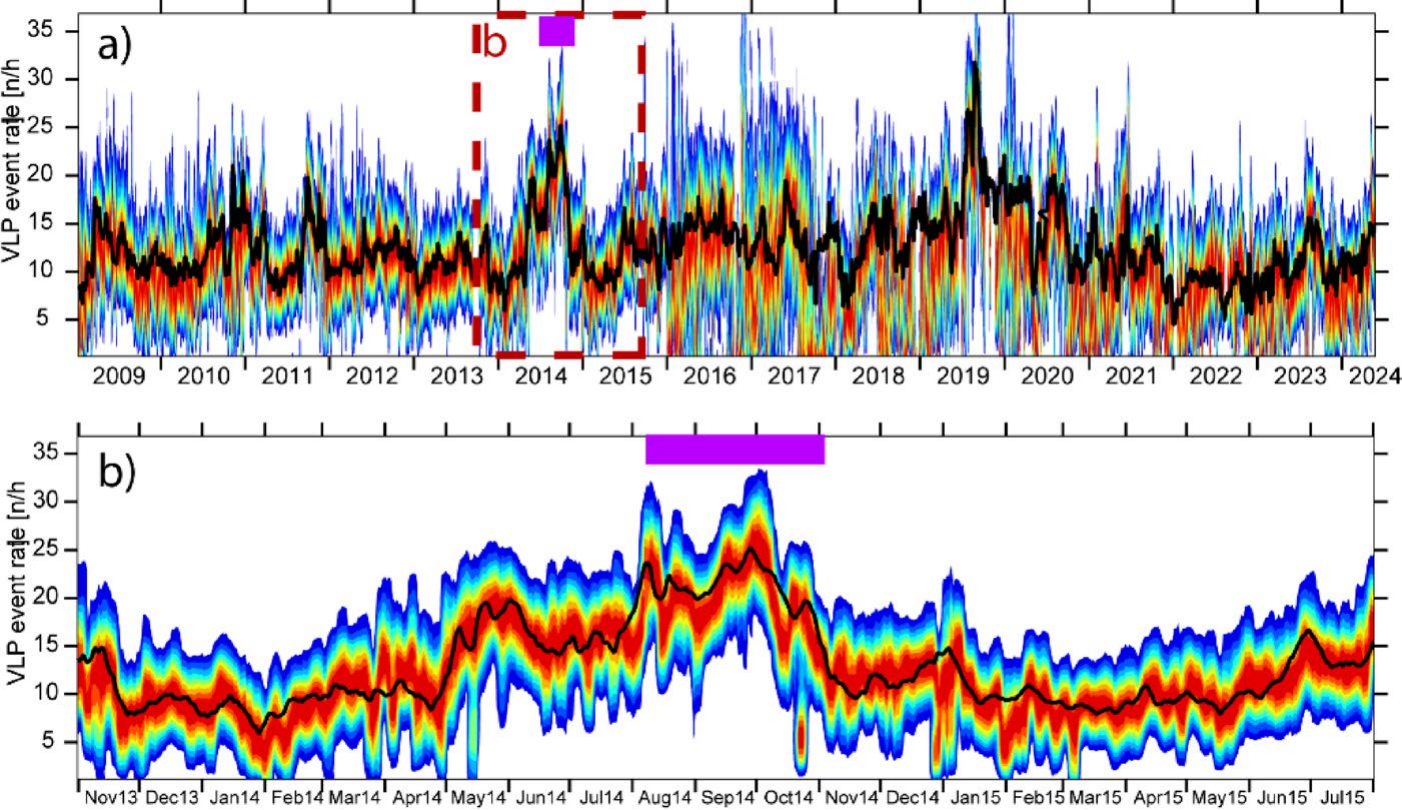
(**a**) Daily VLP occurrence rate from January 2009 to May 2024, as estimated by the detection algorithm and compared with the manually determined rate from the seismic monitoring room (black line). In the 2D histogram, red areas indicate the highest event density, while blue areas indicate the lowest. (**b**) Close-up of the 2013–2015 period, showing the increasing VLP rate leading up to the onset of the 2014 eruption (highlighted by a purple bar), followed by a gradual decline in VLP activity back to pre-eruptive levels. The black line corresponds to the manually assessed event rate.

**Fig. 8:**
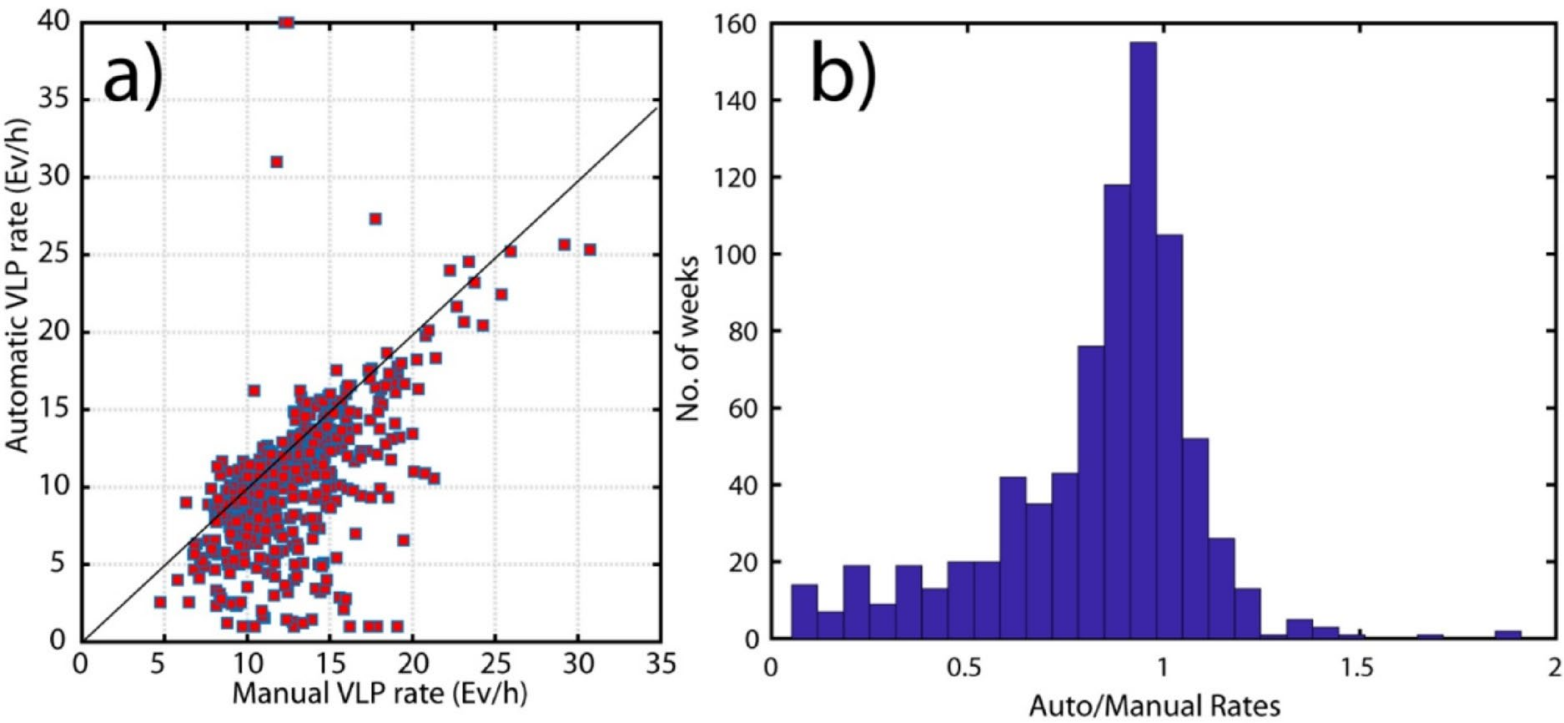
In panel (**a**), the data show a general 1:1 correlation, indicated by the black line, along with a systematic underestimation of rates by the automatic method. The Pearson correlation coefficient is *R* = 0.6. In panel (**b**), we present the histogram of the events from panel (**a**), showing that most of the rates are close to 1.

Once the algorithm was calibrated and validated on the 2007 dataset, we extended our analysis over a longer time window of 16 years, from January 2009 to May 2024 (Fig. 7). Unfortunately, for this extended period, manual picks from the INGV Monitoring Center are not available, but only daily VLP detection rates are provided. We therefore focused on this parameter to test the algorithm’s ability to capture variations in VLP activity, even during long periods of ordinary Strombolian activity, which largely characterized this timeframe. However, the analysis window also includes two significant eruptive periods: August–November 2014^23,41,42^ and July–August 2019^25,43^. Similarly to the test performed on the 2007 dataset (Fig. 6), the VLP rate provided by the automatic method results from combining the rates computed at individual stations, selecting the station with the highest F1-score as the reference. As a result, the rate is primarily representative of station STRA. However, when STRA was not operational, the rate from the best-performing available station in terms of F1-score was used instead (Table 3). Furthermore, since the automatic detection rates are provided on an hourly basis, we computed the daily distribution of these hourly rates to compare them with the average daily VLP counts reported by the seismic monitoring center (Fig. 7). The most representative value for each day is taken as the mode of the distribution. Additionally, the distribution provides an estimate of the uncertainty associated with the daily rate estimation. Both the automatic and manual VLP counts mostly range between 5 and 30 events per hour, showing peak values during the effusive eruptive periods of 2014 and 2019 (Fig. 7). Fluctuations in the parameter, even during non-eruptive activity, appear consistent between the two methods, confirming the reliability of the algorithm over multi-year timescales. A Pearson correlation coefficient of 0.6 is observed between the two methods (Fig. 8), with most of the data points aligning along the expected 1:1 relationship when plotted against each other. However, Fig. 8 also reveals a tendency of the automatic method to underestimate the VLP counts compared to the manual picks. This bias may be explained by the presence of high noise levels in the VLP frequency band, particularly during periods of adverse weather and strong sea wave activity, which reduces the detectability of VLP events by the automatic method. In contrast, manual analysts tend to mitigate this issue by focusing on quieter time windows within the day when wave-induced noise is lower. There are also a few isolated days when the manual method reports significantly higher counts than the automatic one, which are probably due to an anomalous occurrence of false positive events which need of further investigations (Fig. S1,S2,S5,S6).

## Discussion and conclusions

The analysis conducted in this study highlights the effectiveness of our PAS-based automatic detection algorithm for VLP seismicity at Stromboli, validated through comparison with manually derived dataset. The CM evaluation quantifies the algorithm’s accuracy in detecting and characterizing VLP events. This accuracy is further supported by a multi-scale comparison, ranging from a detailed one-year dataset (2007) to a long-term assessment spanning 16 years (2009–2024), yielding a Pearson correlation coefficient (R) between 0.6 and 0.76 between automatic and manual detection rates. These results demonstrated the robustness of the method in capturing long-term variations in VLP activity, despite some discrepancies observed during periods of particularly high rate and very low-amplitude VLP activity (Fig. 6). The best filtering parameters were established through a station-wise tuning approach using the Optuna framework^22^, which allowed us to optimize precision, minimizing false positives. The optimization process was deliberately oriented toward maximizing precision rather than recall. This choice was made to ensure the reliability of event detection, even at the cost of missing a greater number of events (Fig. 8). As a result, the VLP rates may be slightly underestimated, but the accuracy of source-related parameters, such as azimuth and incidence, is improved. The method proves especially useful for continuous monitoring during periods of volcanic unrest, when network performance may be degraded by eruptive activity or external noise sources. Although the developed algorithm filters out most signals, spurious events still remain in the dataset, as indicated by a precision that never exceeds 0.81 (Table 2). Overall, the results indicate that stations such as STRA, STR1, and STR8 consistently achieve the highest precision scores. These stations are located on the eastern flank of the island, sheltered from the prevailing westerly winds (Fig. 1). In contrast, other stations, such as STR5, exhibited high precision but less reliable F1-scores, likely due to greater exposure and higher ambient noise levels. Overall, this method offers a practical solution for near-real-time monitoring, operating with minimal computational resources and a processing delay of only about 10 min. One of its key advantages lies in its ease of implementation: the algorithm can be deployed using a Conda environment, making it accessible even to users without advanced computational infrastructure. Its ability to efficiently process long-term seismic records without requiring high-performance computing underscores its suitability for real-time applications, particularly in resource-constrained or remote settings where streamlined, reliable tools are essential. We furthermore provide the code on Github (see Data Availability Statement). This study allowed also to build a comprehensive seismic database of VLP waveforms that not only provides valuable insights into volcanic behavior but also serves as a foundational dataset for the development of AI-based real-time monitoring systems, potentially including unsupervised approaches^44,45^. Such systems could further enhance detection capabilities, enabling faster responses to seismic events and more accurate eruption forecasting. The integration of advanced machine learning techniques and optimized sensor network coverage could refine these models, increase detection precision, and reduce false negatives^46^.The continued development and operational implementation of automatic VLP detection, combined with AI-driven real-time monitoring systems, will play a critical role in strengthening early warning capabilities and mitigating volcanic risk^47^. The creation of this database^48^ also enabled a long-term analysis of VLP event azimuth and incidence, offering valuable insights into the evolution of volcanic processes, as they relate to the signal’s propagation direction and thus the relative position of the source with respect to the station. By incorporating these polarization features, our catalog offers a more complete characterization of VLP events, enhancing both the accuracy of volcanic monitoring and the interpretation of Stromboli’s long-term seismic behavior. In line with previous findings, our results confirm that polarization parameters are sensitive indicators of magma migration within the conduit. During the 2007 eruption, a sudden shift in polarization parameters, consistent across multiple stations, was observed and interpreted as the draining of the shallow magma reservoir^40^. This pattern aligns with the well-documented eruptive sequence of Stromboli and underscores the sensitivity of VLP signals to changes in magmatic dynamics^2–4^. Moreover, our findings reinforce previous observations that fluctuations in VLP rates sometimes precede major eruptive events, highlighting the importance of integrating automated VLP detection into early warning systems.

## Supplementary Information

Below is the link to the electronic supplementary material.

Supplementary Material 1

## Data Availability

The code described in this article is publicly available on GitHub at [https://github.com/SergioGammaldi/VLPBEATs], while the dataset can be accessed at reference (48).
