# Supplementary material for: A near real-time framework for monitoring very-long-period signals at volcanoes
Source: Sci Rep. 2025 Nov 24;15:41626. doi: 10.1038/s41598-025-25636-7 (PMC12644686; doi:10.1038/s41598-025-25636-7)

# **Figure S1**: The figure shows an example of seismograms with a storm and the detected event in the red line. From the top to the bottom we show: the vertical, the North-South, and the East-West components.


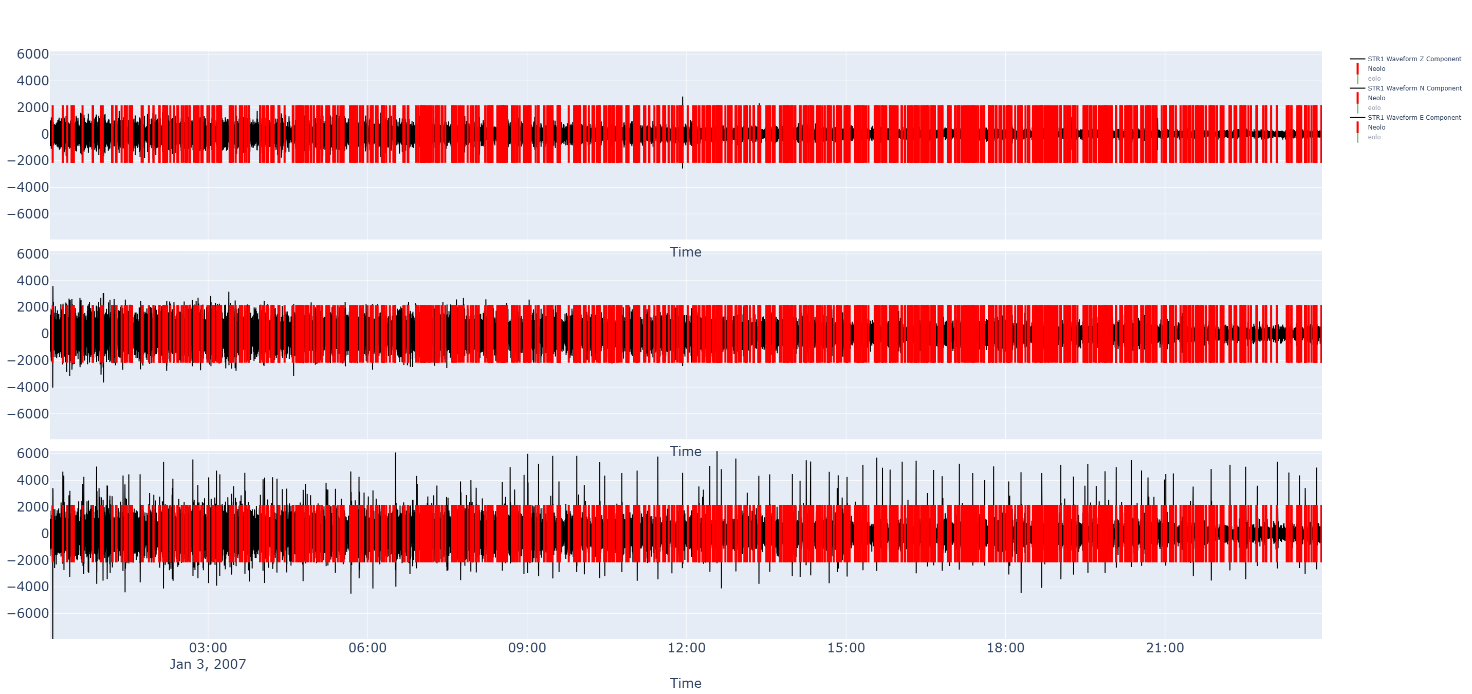


# **Figure S2**: The figure shows an example of seismograms with teleseismic and the detected event in red line. From the top to the bottom we show: the vertical, the North-South, and the East-West components.


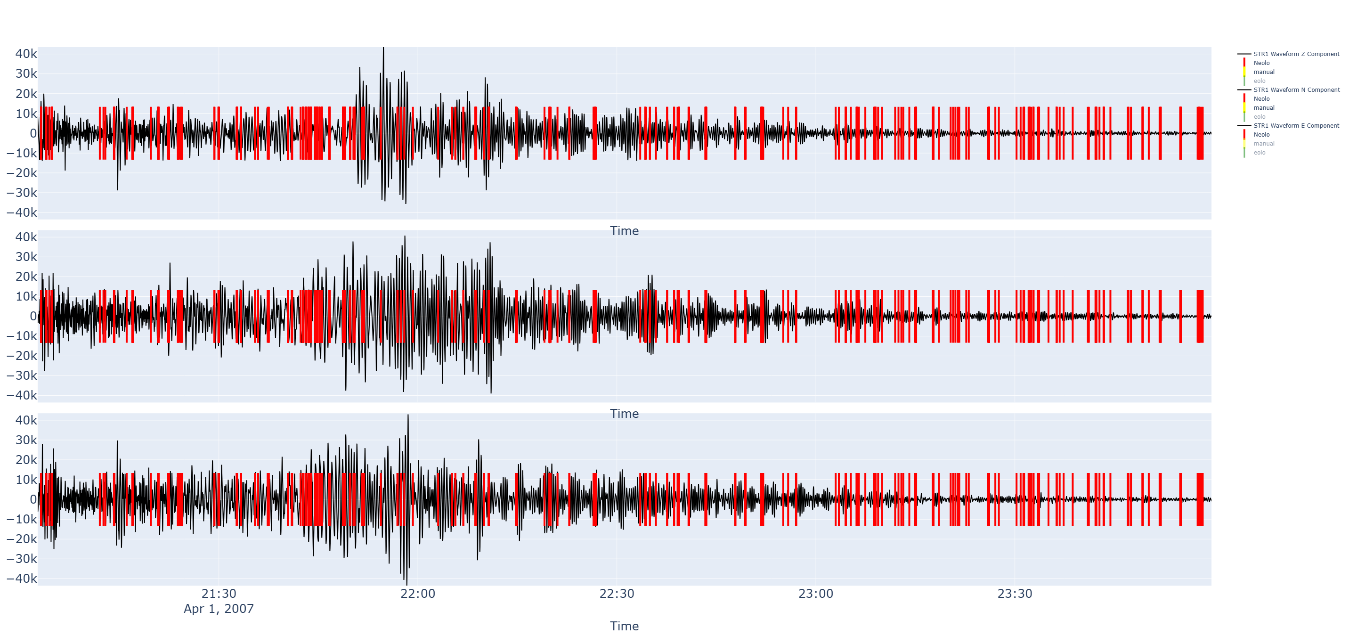


# **Figure S3**: The image shows the three time-variant frequencies along the three signal components. The first row is related to the Vertical component, while below are shown North-South and the East-West components. The color bar enhances the frequency amplitudes of the spectrum.
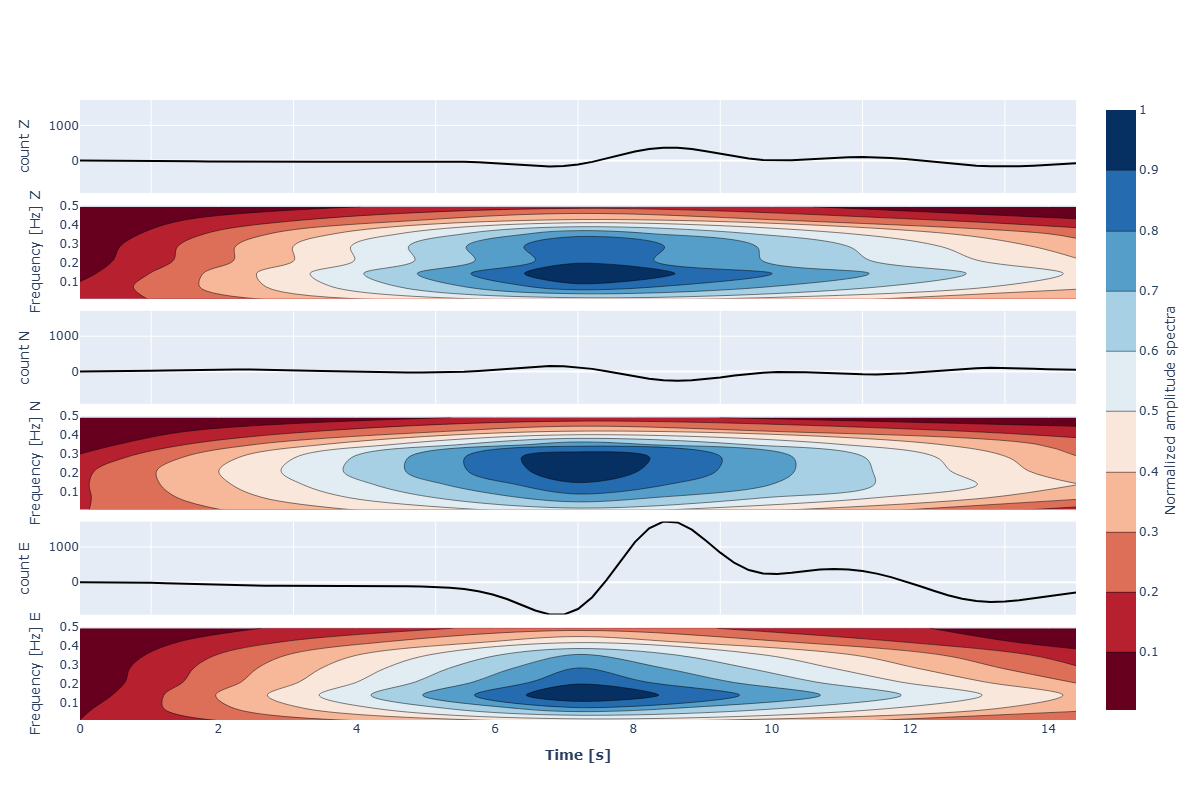


# **Figure S4**: F1 Score vs. Iteration for All Stations. Each subplot shows the evolution of the F1-score over Optuna trial iterations for a specific station. The blue line with markers represents the raw F1 score for each trial, while the red line indicates a smoothed trend (5-trial moving average). The plots demonstrate that performance generally improves rapidly in early iterations and stabilizes thereafter, suggesting convergence of the optimization. Most stations exhibit asymptotic behavior, indicating that further trials yield diminishing returns.


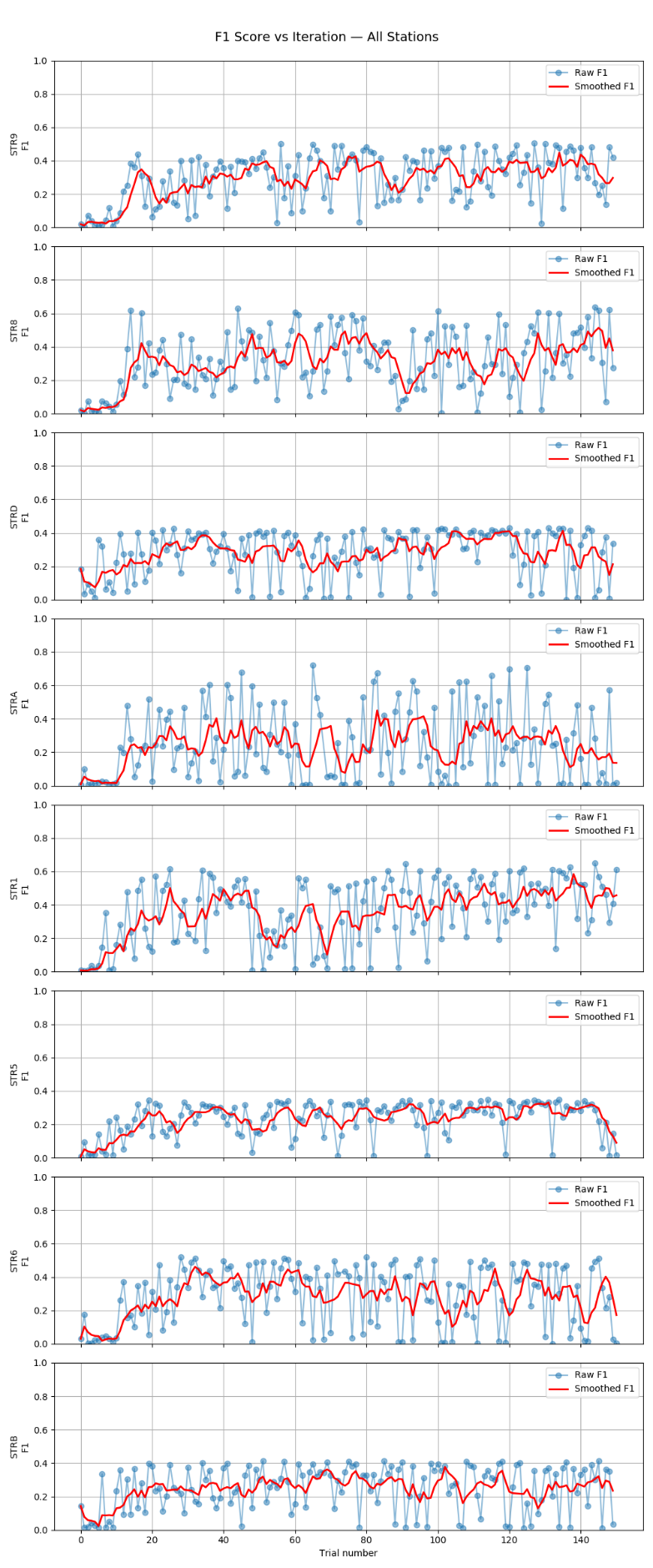


# **Figure S5**: The figure shows an example of seismograms with a storm and the detected event in the red line after having applied the std filter. From the top to the bottom we show: the vertical, the North-South, and the East-West components.


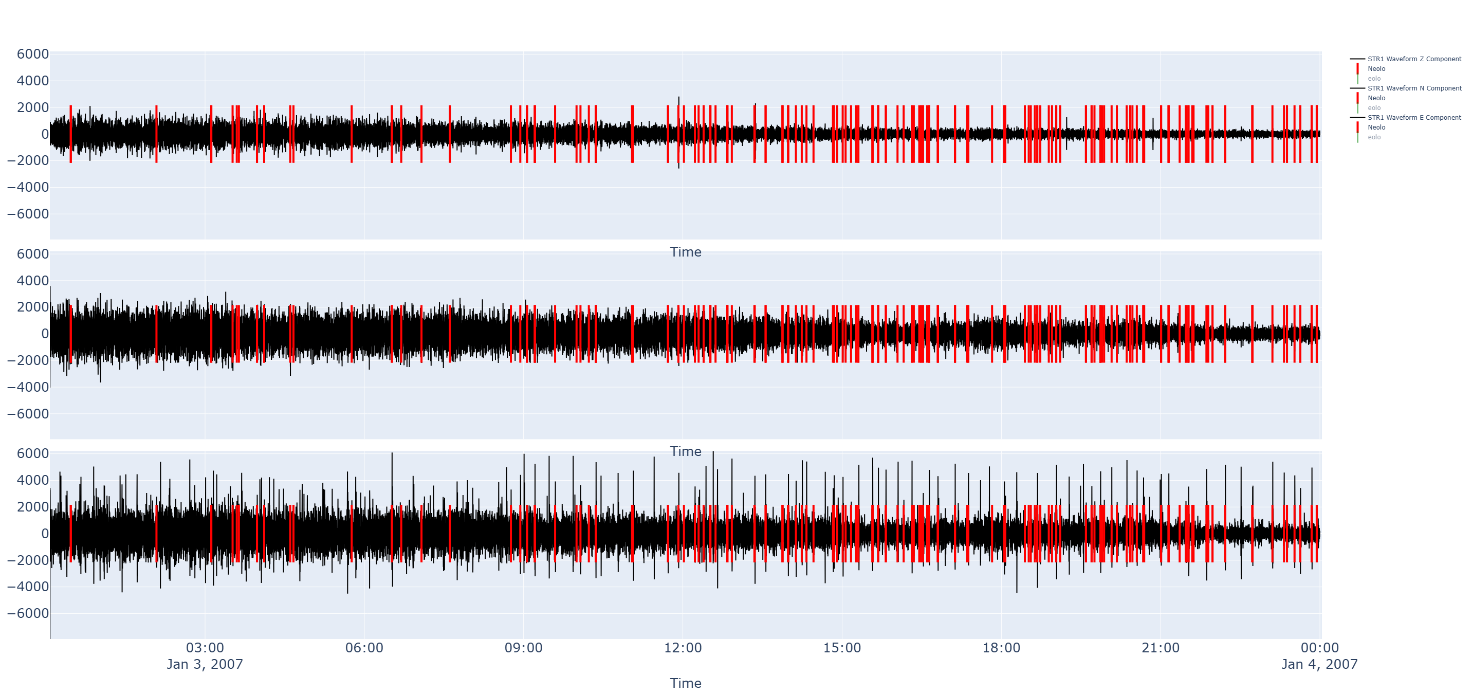


# **Figure S6**: The figure shows an example of seismograms with teleseismic and the detected event in red line after having applied the std filter. From the top to the bottom we show: the vertical, the North-South, and the East-West components.


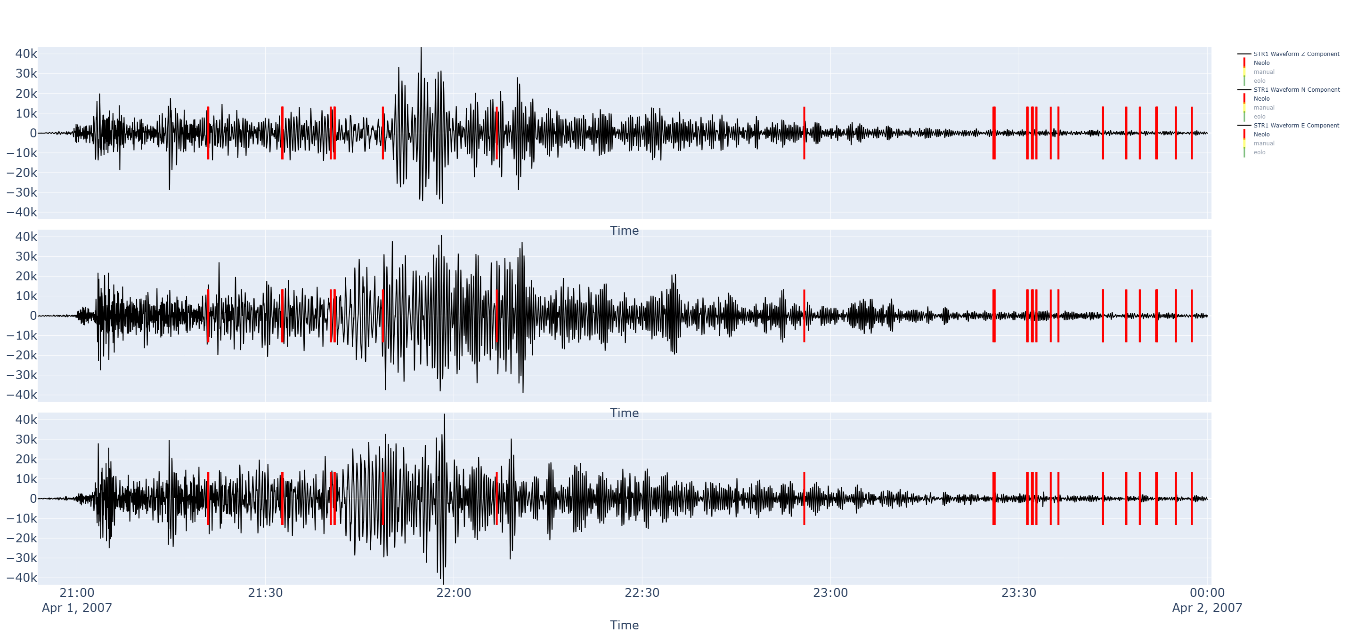


# **Figure S7**: Correlation rate between manual and automatic rate along the 2007.


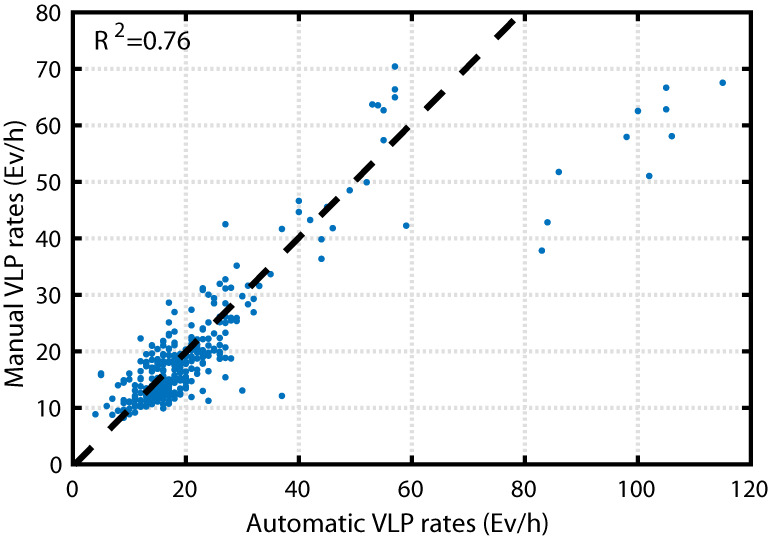


# **Table S1**: Polarization attributes formulas and references.


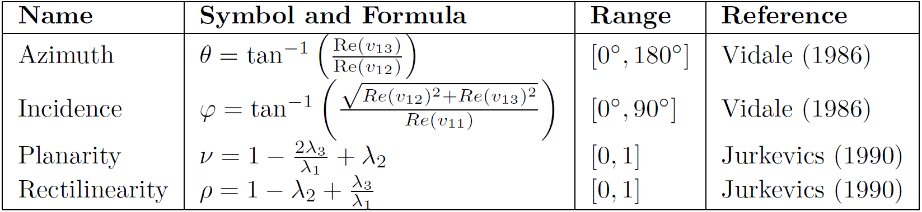

Supplement: Supplementary file 1 — Supplementary Material 1 [file 41598_2025_25636_MOESM1_ESM.docx]
